# Group-training of rams at puberty for artificial vagina-mediated semen collection and its influence on semen quality and sexual behavior

**DOI:** 10.1590/1984-3143-AR2022-0051

**Published:** 2023-04-21

**Authors:** Majdi Ahmed Bahadi, Mohammed Abdo Al-Badwi, Emad Mohammed Samara, Khalid Ahmed Abdoun, Ibrahim Abdullah Alhidary, Ahmed Abraham Al-Haidary

**Affiliations:** 1 Department of Animal Production, College of Food and Agriculture Sciences, King Saud University, Riyadh, Saudi Arabia

**Keywords:** artificial insemination, CASA, flehmen, libido, puberty

## Abstract

There is a paucity of information with respect to group-training for artificial vagina and its influence on semen characteristics and sexual behavior of young untrained rams. A total of 18 healthy Najdi rams (with an initial body weight of 40-45 Kg and 7-8 month-old) were consequently used herein to test the usefulness of group-training for artificial vagina-mediated semen collection during the breeding season. Rams were randomly segregated into three groups (*n* = 6 rams per protocol), and the whole experiment was lasted for 10 weeks. The 1^st^ group was subjected to a training protocol where one untrained ram was placed for 20 min with a teaser ewe, while the 2^nd^ group were subjected to a protocol where one untrained ram was placed for 20 min with one trained ram and a teaser ewe, whereas the 3^rd^ group were subjected to a protocol where three untrained rams were placed for 20 min with one trained ram and a teaser ewe. The obtained results clearly (*P* < 0.05) showed that training young rams in group has increased their sperm concentration and sexual stimulation, shortened the period of their training time, and descriptively had a complete training efficiency. The sexual stimulation of young untrained rams was intensified by the competition between rams in the co-presence of a trained ram. Collectively, these data may suggest that group-training of rams at puberty is a better protocol for AV-mediated semen collection compared to individual training. Some shortcomings were noted herein, but research dealing with this subject may very well improve the reproductive performance of young untrained rams.

## Introduction

For electroejaculator- or artificial vagina (AV)-mediated semen collection, young rams during puberty require previous training in order to habitualize the whole process of taming and the presence of the collector (Stafford, 1995; Mulu et al., 2018; McMurran, 2020). There are several approaches to train rams for AV-mediated semen collection such as using estrual / non-estrual ewe, hormone-treated ewes, and mounting an inanimate object such as manikin/dolly (Flores et al., 2005; Ambrosi et al., 2018). However, training rams using these approaches is usually costly (Stafford, 1995). Therefore, looking for an alternative and efficient training protocol to improve and enhance the reproductive performance of rams constitute an important issue. As a matter of fact, there is a scarcity of information with respect to such type of protocols and their influence on semen characteristics.

Besides, rams during sexual intercourse usually show several behaviors that includes -but not limited to- flehmen response by elevating the head and retracting the upper lip, gargling, sniffing the genital area of the ewe, sensing ewe’s urine, repeatedly pawing nuzzling, licking, nibbling at ewe’s flank while standing behind, and occasionally vocalize before/during copulation (Perkins and Roselli, 2007; Fonsêca et al., 2013; Santos et al., 2015). Under a training process of AV-mediated semen collection, live evaluations of early sexual behavior for rams are rare (Perkins and Roselli, 2007).

Consequently, the aim of this experiment was to develop and test different protocols in order to train young rams for AV-mediated semen collection. We hypothesized that mixing untrained and experienced rams at the time of training may increase the sexual stimulation of untrained rams and can shorten the period of their training time, which subsequently may improve their reproductive performance.

## Methods

### Location

The current experiment was conducted at the experimental farm animal station affiliated to the Department of Animal Production, College of Food and Agriculture Sciences, King Saud University (24°48'20.8”N, 46°31'14.2”E). Animal use for scientific purposes as well as experimental procedures described herein were in accordance with the Animal Welfare Practices Act and approved by the Research Ethics Committee, King Saud University (Process number: KSU-21-66).

### Animals and management

Beside one teaser (multi-parous, not treated with hormones, and 3 years of age) Najdi ewe and four mature (one year of age) trained Najdi rams, a total of 18 healthy young untrained Najdi rams (with an initial body weight of 40-45 Kg and 7-8 month-old) were used herein between December of 2020 and March of 2021 (~ during the breeding season). After weaning at two month of age, these rams were separated from their dams as lambs, and had no sexual experience prior to this experiment. All rams were ear tagged, vaccinated against certain infectious diseases and received multivitamins and prophylactic doses of anthelmintic, before the commencement of the experiment. Rams were fed daily at 8:30h throughout the experiment at 3.0% of their initial body weight on a commercial pelleted-complete diet containing 11.30 MJ/kg ME and 13.00% CP on DM basis (ALWAFI-ARASCO, KSA; feed ingredients included: barley, wheat, palm kernel meal, soybean hulls, wheat bran, alfalfa, salt, limestone, molasses, acid buffer and commercial premix). In addition, fresh water and mineral block were available *ad libitum*. The recorded meteorological indices (ambient temperature= 22.10 ± 0.25 °C, relative humidity= 28.28 ± 0.46%, and temperature-humidity index= 65.99 ± 0.26 Units) clearly indicated that rams were within their thermo-neutral condition throughout the experiment (Rout et al., 2018).

### Experimental design

The entire experiment was lasted for 10 weeks; the first 6 weeks were served as a preparatory phase during which 4 mature rams were trained for mating and semen collection until their eligibility for sexual intercourse and semen collection via AV was assured. During a 4-week experimental phase, on the other hand, three training protocol were weekly used to train young rams for AV-mediated semen collection. Before the commencement of semen collection, rams were randomly segregated into three groups (*n* = 6 rams per protocol). The 1^st^ group was subjected to a training protocol (named P1) where one untrained ram was placed for 20 min with the teaser ewe (consider herein as a control group), while the 2^nd^ group were subjected to a training protocol (named P2) where one untrained ram was placed for 20 min with one trained ram and the teaser ewe, whereas the 3^rd^ group were subjected to a training protocol (named P3) where three untrained rams were placed for 20 min with one trained ram and the teaser ewe. Rams were placed with the ewe according to the aforementioned protocols and their semen samples were evaluated using a computer-assisted sperm analysis (CASA), while their sexual behavior was monitored and evaluated with the help of a video camera. It is noteworthy that rams had received a small portion of Alfalfa (*Medicago sativa*) every time they effectively completed a mounting, in order to facilitate conditioning and reinforcement.

### Experimental measurements

One semen sample was collected every week from each ram in the morning using the AV method while mounting the restrained teaser ewe, in which a warm water (around 40-60°C) and air were introduced between an outer casing and soft inner sleeve to simulate the vaginal condition, a petrolatum was used to lubricate the end where penis’s intromission occurs, and a rubber funnel terminating at a transparent graduated tube measuring to the nearest 0.10 mL was attached at the opposite end to collect the ejaculated semen.

Ejaculates were immediately immersed in a water bath (37.00 ± 2.00 °C) after collection, and then transported within approximately 15 min of collection to the laboratory for analysis by CASA using Sperm Class Analyzer (SCA) software (SCA^®^ 2002, Microptic, Barcelona, Spain). The setup parameters for sheep semen were pre-established according to the manufacturer’s instructions. For every successfully collected semen sample, at least 10 fields were examined for the following parameters: total sperm concentration (SC, 10^6^/mL), percentage of progressively motile spermatozoa (PMS, motility > 20 μm/s), percentage of non-progressively motile spermatozoa (NPMS, motility < 20 μm/s), percentage of immotile spermatozoa (IS, motility < 5 μm/s), velocity of a curved line (VCL, μm/s), velocity of a straight line (VSL, μm/s), velocity of the average path (VAP, μm/s), linearity (LIN = VSL/VCL), straightness (STR = VSL/VAP), wobble (WOB = VAP/VCL), amplitude of lateral head displacement (ALH, μm), and beat cross frequency (BCF, Hertz).

Regarding the evaluation of rams’ sexual behavior, a camera (coupled with a voice recorder) was first used to document all observations, and then the recorded videos were screened and analyzed according to the method of Ambrosi et al. (2018). In brief, the teaser ewe was restrained in one spot before rams entered the training area. Recordings of 15-min period (by two observers each stayed in a corner) were started once the rams entered the area and seen the ewe. When a ram mounted the ewe, the collector technician rapidly approached and collected the ejaculated semen using an AV. However, once the 15-min period was finished, the ram was removed from the training area (regardless of the mounting and collection). Recorded videos were thereafter assessed for the following parameters: sniffing (nS, times), duration of sniffing the ewe (tS, sec), flehmen expression (FE, times), duration of flehmen (tFE, sec), pushing the ewe (P, times), pawing the side of the ewe with chin resting on it (PaCR, times), foreleg kicking (FK, times), tongue flicking (TF, times), mounting without intromission (MWOI, times), duration of mounting without intromission (tMWOI, sec), duration of mounting with ejaculation (tMWE, sec), and completed time (CT: the total time from the approaching to the completed mount, sec). Furthermore, the weekly training efficiency (WE, %) of each protocol was calculated as follow: [number of rams mounted with ejaculation / total number of animals * 100].

### Statistical analysis

Experimental data were analyzed as a complete randomized design using the PROC GLM procedure of SAS 9.4 (SAS Institute Inc., Cary, NC) to determine the differences in all parameters as a function of the fixed effect of different training protocols. The descriptive statistics for all parameters were calculated using the PROC MEANS method. Data were then subjected to repeated measure ANOVA, where means showing significant differences were tested using the PDIFF option. In fact, statistical means were compared, thereafter, using least significant difference (LSD) test. Unless otherwise indicated, the probability value -which denotes statistical significance- was set at *P* < 0.05.

## Results

The obtained findings of the physical analysis of semen samples illustrated that the overall means of both SC and ALH were increased (*P* < 0.05), while that overall means of both VSL and BCF were decreased (*P* < 0.05) with the shift from P1 to P3 (Table 1). Notably, however, no difference (*P* ≥ 0.05) was observed between the applied protocols in the rest of the seminal kinematic parameters (Table 1).

**Table 1:** Influence of group-training of rams at puberty on physical characteristics of semen collected via artificial vagina method during the breeding season (*n* = 6 rams per protocol).

**Parameters** **1**	**Training protocol** **2**			**SEM**	***P* value**
	**P1**	**P2**	**P3**		
***SC (x10^6^/mL)***	3261.57^b^	4234.99^ab^	5290.35^a^	589.47	0.034
***PMS (%)***	34.94	33.33	28.34	2.58	0.330
***NPMS (%)***	43.68	45.32	43.66	3.67	0.850
***IS (%)***	21.38	21.35	28.00	2.89	0.170
***VCL (μm/s)***	72.95	69.47	65.12	3.33	0.530
***VSL (μm/s)***	52.63^a^	42.65^b^	38.37^b^	3.52	0.010
***VAP (μm/s)***	58.62	56.88	51.87	3.73	0.730
***LIN (%)***	0.51	0.49	0.47	0.18	0.430
***STR (%)***	0.61	0.61	0.61	0.18	0.810
***WOB (%)***	0.07	0.68	0.67	0.16	0.870
***ALH (%)***	2.40^c^	2.73^b^	7.78^a^	0.11	0.002
***BCF (%)***	2.94^a^	2.40^b^	2.59^ab^	0.15	0.040

^1^SC: Sperm concentration; PMSP: progressively motile spermatozoa; NPMS: non-progressively motile spermatozoa (%); IS: immotile spermatozoa; VCL: velocity of curved line; VSL: velocity of straight line; VAP: velocity of average path; LIN: linearity; STR: straightness; WOB: wobble; ALH: amplitude of lateral head displacement; and BCF: beat cross frequency (see text for details). ^2^P1: one untrained ram was placed for 20 min with a teaser ewe (consider herein as a control group); P2: one untrained ram was placed for 20 min with one trained ram and a teaser ewe; and P3: three untrained rams were placed for 20 min with one trained ram and a teaser ewe. ^a-c^ Means within the same row bearing different superscripts are significantly different at *P* < 0.05.

**Figure 1:**
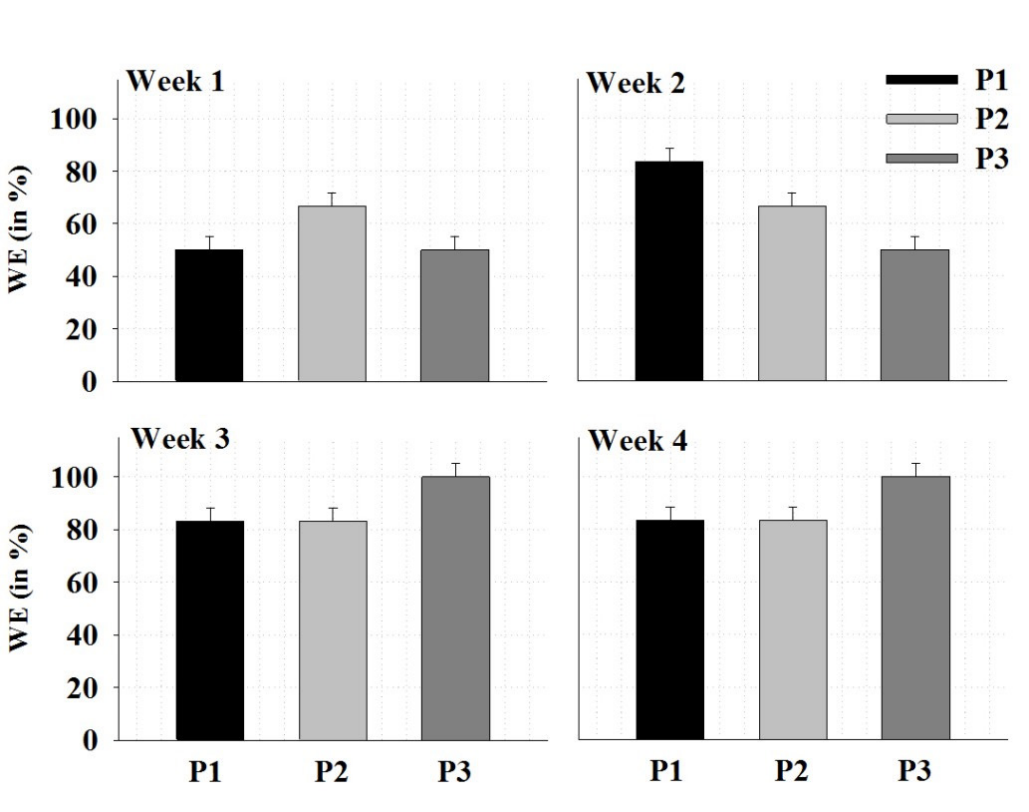
Weekly efficiency (WE, %) of three training protocols used for artificial vagina-mediated semen collection (*n* = 6 rams per protocol) during the breeding season. P1: one untrained ram was placed for 20 min with a teaser ewe (consider herein as a control group); P2: one untrained ram was placed for 20 min with one trained ram and a teaser ewe; and P3: three untrained rams were placed for 20 min with one trained ram and a teaser ewe.

The influence of group-training of young rams for AV-mediated semen collection on their sexual behavior is presented in Table 2 and Figure 1. When P3 rams were compared to their counterparts of P1 rams, results revealed that P3 had exhibited (*P* < 0.05) higher values of nS, TF, MWOI, and CT, but lower (*P* < 0.05) value of FE (Table 2). Generally speaking, P2 rams have showed moderate sexual behavior compared to other protocol’s rams (Table 2). Furthermore, group-training of rams for AV-mediated semen collection at puberty descriptively has a noticeable influence on the calculated WE of rams. In the P1 rams, the WE started from 50% (3 animals out of 6) at the 1^st^ week, then reached 83% (5 animals out of 6) at the 2^nd^ week, and continued at that level till the end of the experiment, whereas the WE of the P2 rams started slightly higher from 67% (4 animals out of 6) at the 1^st^ week but continued at that level for a 2^nd^ week, then reached 83% (5 animals out of 6) at the 3^rd^ week till the end. Meanwhile, the WE of the P3 rams started from 50% (3 animals out of 6) at the 1^st^ week and continued at that level for a 2^nd^ week, then reached a complete training efficiency (100%, 6 animals out of 6) at the 3^rd^ week and continued at that level till the end of the experiment (Figure 1).

**Table 2:** Influence of group-training of rams at puberty for artificial vagina-mediated semen collection during the breeding season on their sexual behavior (*n* = 6 rams per protocol).

**Parameters** **1**	**Training protocol** **2**			**SEM**	***P* value**
	**P1**	**P2**	**P3**		
**nS (*Times*)**	3.29^ab^	2.33^b^	4.58^a^	0.63	0.05
**tS (*sec*)**	7.55	7.93	6.55	1.59	0.24
**FE (*Times*)**	0.71^a^	0.09^b^	0.17^ab^	0.20	0.55
**tFE (*sec*)**	12.13	19.00	7.25	6.81	0.40
**P (*Times*)**	0.83	0.92	1.21	0.20	0.38
**PaCR (*Times*)**	1.33	1.38	1.42	0.31	0.98
**FK (*Times*)**	1.42	0.83	1.96	0.49	0.27
**TF (*Times*)**	1.75^b^	3.25^ab^	4.08^a^	0.78	0.11
**MWOI (*Times*)**	0.83^b^	1.04^ab^	1.79^a^	0.31	0.07
**tMWOI (*sec*)**	5.08	5.68	7.06	2.23	0.88
**tMWE (*sec*)**	15.65	11.89	8.94	5.56	0.85
**CT (*sec*)**	45.91^b^	143.42^ab^	166.96^a^	31.49	0.02

^1^nS: sniffing the ewe; tS: duration of sniffing; FE: flehmen expression; tFE: duration of flehmen; P: pushing the ewe; PaCR: pawing the side of the ewe with chin resting on; FK: foreleg kicking; TF: tongue flicking; MWOI: mounting without intromission; tMWOI: duration of mounting without intromission; MWE: mounting with ejaculation; tMWE: duration of mounting with ejaculation; and CT: completed time (see text for details). ^2^P1: one untrained ram was placed for 20 min with a teaser ewe (consider herein as a control group); P2: one untrained ram was placed for 20 min with one trained ram and a teaser ewe; and P3: three untrained rams were placed for 20 min with one trained ram and a teaser ewe. ^ab^ Means within the same row bearing different superscripts are significantly different at *P* < 0.05.

## Discussion

Finding alternative and efficient training protocols to improve and enhance the reproductive performance of rams constitute -as previously highlighted- an important issue. In fact, little data exists regarding group-training for AV and its influence on semen characteristics and sexual behavior of young untrained rams. As a result, the present experiment was conducted to test three protocols in order to train young rams for AV-mediated semen collection.

According to the physical characteristics of semen samples, our results indicated that group-trained rams (P2 and P3) had slightly lower seminal quality than individually trained rams (P1; Table 1). Despite the increase in seminal SC value (measures the total sperm concentration in the ejaculate), shifting from P1 to P3 failed to demonstrate any benefits to the rest of the seminal kinematic parameters. In fact, semen samples collected from group-trained rams -compared to individually-trained rams- had higher value of ALH (measures the magnitude of the lateral displacement of a sperm head about its average path), and lower values of both VSL (measures the velocity of a sperm head on a time-average basis along the straight line between its first and last detected position) and BCF (measures the average rate at which the actual sperm trajectory crosses the velocity of the average path). Ideally, sperm concentration is critical to ensure a higher number of spermatozoa that traveled long distances and at higher speeds (Gilmore, et al., 2021). However, several authors showed that strong correlations do exist between sperm swimming velocity and fertility, where sperms with greater mobility are indicative of increased sperm metabolism or working organelles (Gerris and Khan, 1987; Amann, 1989; Amann and Hammerstedt, 1993; Krause, 1995; Malo et al., 2005; Kathiravan et al.,2008; Broekhuijse et al., 2012; Nagy et al., 2015; Campanholi et al., 2017). Accordingly, this may suggest that group-training of rams for AV-mediated semen collection at puberty should tentatively be avoided.

To verify, the reproductive performance of these rams was further assessed in the current experiment by recording their sexual behavior. Numerous experiments reported an increase in rams’ libido, because of social and physiological cues, when stimulated by various ways (such as visual, olfactory, and/or full physical contact), or when exposed to other rams been in previous contact with ewes (Maina and Katz, 1999; Fahey et al., 2012; Preston et al., 2012; Carrillo et al., 2014). Interestingly, the present data revealed that P3 rams had exhibited higher values of nS, TF, MWOI, and CT, when compared to their P1 counterparts, while P2 rams showed moderate sexual behavior compared to other protocol’s rams (Table 2). The extremely longer social contact of the grouped rams in P3 might induced a higher stimulation of the pituitary gland to secrete a higher amount of gonadotropin releasing hormone (GnRH), which ultimately stimulated the testes to release more testosterone hormone to peripheral blood and thus a higher libido expression. Actually, these findings are consistent with previous reports stating that higher libido increases nS, TF, MWOI, CT, as well as blood peripheral testosterone hormone, which ultimately might led to more series of epididymal contractile waves and subsequently to semen ejaculates with high SP (Almquist, 1973; Hemsworth and Galloway, 1979; Gonzalez et al., 1991; Prado et al., 2002, 2003; Silvestre et al., 2004; Fahey et al., 2012). Notably, this may therefore explain the descriptively complete training efficiency as well as the early training ability obtained herein for P3 rams compared to other training protocols (Figure 1). This actually represents substantial evidence that lower sexual behaviors might be as a result of weak or short duration of sexual contact time as experienced herein by P1 and/or P2 rams. Coupled with higher SC value (Table 1), it collectively implies that P3 may be a better protocol for AV-mediated semen collection at puberty.

## Conclusions

This experiment is one of the first that investigate the usefulness of group-training of rams during the breeding season for AV-mediated semen collection at puberty on their semen quality and libido. The obtained results plainly showed that training young rams in group may increase their sperm concentration and sexual stimulation, shortened the period of their training time, and descriptively had a complete training efficiency; thereby, suggesting that group-training of young rams is a better protocol for AV-mediated semen collection compared to individual training.

However, this experiment is not without limitations. For instance, information on the levels of dead and live spermatozoa as well as sexual hormones under such conditions should all be assessed in future experiments. Additionally, there may be a need to conduct a broader experiment to include more animals as well as to determine the influence of other factors such as the breed and age of trained and untrained rams. The feasibility and freezability of the first collected semen samples of young untrained rams should be investigated, as well.
